# In situ, antibody-independent, and multiplexed characterization of amyloid plaques by MALDI MS/MS imaging using iprm-PASEF

**DOI:** 10.1007/s00216-025-06277-1

**Published:** 2026-01-14

**Authors:** Larissa Chiara Meyer, Mujia Jenny Li, Nadine Meier, Beate Vollmer-Kary, Tobias Feilen, Julie Audebert, Konrad Kurowski, Stephan Singer, Peter Bronsert, Melanie Christine Föll, Oliver Schilling

**Affiliations:** 1 Institute for Surgical Pathology, Faculty of Medicine, University Medical Centre Freiburg, University of Freiburg, Freiburg, Germany; 2 Faculty of Biology, University of Freiburg, Freiburg, Germany; 3 Institute for Pharmaceutical Sciences, University of Freiburg, Freiburg, Germany; 4 Institute of Pathology, University of Tübingen, Tübingen, Germany; 5 Core Facility for Histopathology and Digital Pathology, Medical Center, University of Freiburg, Freiburg, Germany; 6 German Cancer Consortium (DKTK), German Cancer Research Center (DKFZ), Heidelberg, Germany

**Keywords:** Amyloidosis, iprm-PASEF, MALDI imaging, MS/MS imaging, TIMS, Peptide imaging, Spatial proteomics

## Abstract

Amyloidosis collectively describes a heterogeneous group of protein aggregation-based diseases involving the misfolding and extracellular accumulation of fibril-forming amyloid proteins. Diagnosing amyloidosis is difficult due to its many subtypes (e.g., AA, AL, ATTR), with varying symptoms. Current diagnosis often involves Congo red staining, but it has limitations in quantification and specificity. A novel method called iprm-PASEF exploits MALDI imaging and offers a faster, spatially resolved, antibody-independent technique for identifying peptides while preserving tissue structure. In this study, iprm-PASEF was used to further evaluate its applicability on amyloidosis. FFPE slides of an amyloidosis TMA including biopsies of 18 amyloidosis-positive tissues were prepared for tryptic peptide MALDI imaging. An initial MALDI TIMS MS1 measurement was performed, followed by the manual generation of a precursor list containing mass-to-charge ratios and ion mobility windows. In a second iprm-PASEF measurement, the selected precursors are analyzed in a multiplexed MALDI MS/MS mode. Peptide identification was achieved through peptide-to-spectrum matching using MASCOT. Within the course of this study, we characterized an amyloidosis TMA consisting of AA, AL, and ATTR amyloidosis diseased tissue with MALDI imaging of tryptic peptides. We successfully identified eight amyloidosis-related peptides derived from serum amyloid A, vitronectin, apolipoprotein E, serum amyloid P component, and transthyretin receptor in one single iprm-PASEF measurement. Peptide signals mapped to amyloidogenic plaques determined in a Congo red staining. Some of these peptides were specifically found in ATTR and AA amyloidosis. This represents a significant step towards integrating MALDI imaging into the diagnostic process for amyloidosis.

**Graphical Abstract:**

**Figure:**
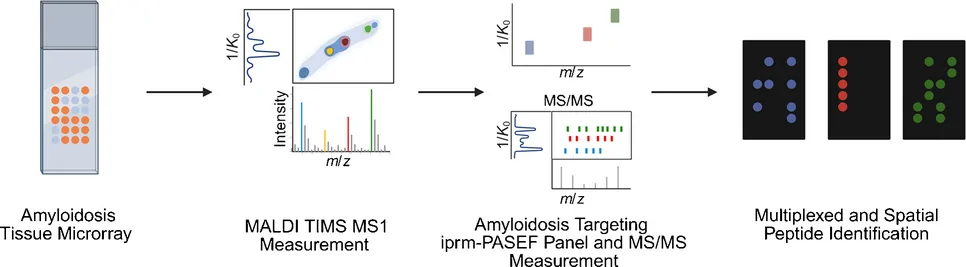

**Supplementary Information:**

The online version contains supplementary material available at https://doi.org/10.1007/s00216-025-06277-1.

## Introduction

Amyloidosis collectively describes a heterogeneous group of protein aggregation-based diseases involving the misfolding and extracellular accumulation of fibril-forming amyloid proteins. The tissue deposition of these fibrils occurs either localized (limited to one organ) or systemically, impairing organ function and, in severe cases, leading to organ failure. Diagnosing amyloidosis remains challenging, as evidence of over 40 amyloid-associated and amyloidogenic proteins leads to a variety of subtypes with heterogeneous symptoms, disease severity, and treatment regimens [1, 2]. The predominant subtypes of amyloidosis include transthyretin (ATTR) amyloidosis (caused by the accumulation of transthyretin monomers), immunoglobulin light chain amyloidosis (AL) (caused by misfolded and/or over-produced and/or incompletely processed immunoglobulin light chains), and the secondary AA amyloidosis (caused by the misfolding and accumulation of serum amyloid A (SAA)). In addition to these subtype-specific proteins, several proteins are found in most amyloid deposits and are often referred to as amyloid-associated proteins. Examples include apolipoprotein E (APOE), serum amyloid P component (SAMP), and vitronectin (VTN) [3–7].

Amyloid plaques in tissue samples (e.g., biopsies) are initially detected by staining with Congo red, an azo dye that stains beta-sheet structures commonly observed in misfolded amyloid proteins. Following a Congo red-positive diagnosis, amyloidosis subtyping is required, involving the identification of the accumulated protein within the Congo red-positive areas, typically by means of antibody-based immunohistochemistry [8] or dissection-based proteomics using liquid chromatography electrospray ionization tandem mass spectrometry (LC–ESI–MS/MS) [3, 9–11]. In the latter, Congo red-positive tissue regions are dissected and subjected to shotgun proteomic analysis for potential detection of tryptic peptides derived from the aforementioned amyloid-associated and amyloidogenic proteins. Both approaches face drawbacks: Immunohistochemistry requires the availability of suitable antibody panels, may consume one slide of limited sample material per staining, and may face limitations with regard to quantification [12]. On the other hand, LC–ESI–MS/MS has the potential to profile the entire set of amyloidogenic proteins with minimal sample input. Yet, extensive sample preparation and ideally laser capture microdissection (LCM) are required to focus on spatially defined, Congo red-positive areas [13–15]. As a result, LC–ESI–MS/MS-based characterization of amyloidosis is laborious.

Matrix-assisted laser desorption/ionization (MALDI) imaging of peptides offers a rapid, spatial, and antibody-independent method to map tryptic peptides derived from amyloid-associated and amyloidogenic proteins. In fact, MALDI imaging has already been applied to amyloidosis in several studies [16–19]. However, in situ and straightforward peptide sequence identification by peptide-spectrum-matching still remains challenging in MALDI imaging. To this end, the aforementioned MALDI imaging studies have used in situ MS2 measurements in a serial manner for selected precursor ions or subsequent LC–ESI–MS/MS analysis with matching of precursor ion masses. However, the first approach defeats the necessity of multiplexed probing for the presence of potential amyloidogenic proteins, while the second approach leads to additional laborious tasks and unreliable peptide identifications.

Recently, we presented imaging parallel reaction monitoring (iprm-) Parallel Accumulation–Serial Fragmentation (PASEF) by utilizing iprm-PASEF-enabled, multiplexed, and MS/MS-based MALDI imaging of peptides that preserves the spatial information on MS2 level [20]. In this method, precursor ions are selected after a classical MS1 imaging scan of the tissue of interest and targeted in a subsequent iprm-PASEF survey, thereby providing the MS2 spectra of the precursor at every single pixel. The iprm-PASEF method exploits TIMS to separate precursors not only by their *m*/*z* value but also by their 1/*K*_0_, thus multiplexing and measuring them in one single MS/MS MALDI imaging run.

In the present proof-of-concept study, we designed a multiplexed and amyloidosis-specific iprm-PASEF panel targeting nine peptides from five amyloidogenic proteins. This panel was applied to a tissue microarray (TMA) containing three different amyloidosis subtypes derived from a total of 18 patients, thus presenting the first application of iprm-PASEF for the in situ characterization of amyloidosis plaques.

## Material and methods

### Sample collection

The Ethics Committee of the University Medical Center Freiburg approved the sample collection and usage for the amyloidosis TMA (Ref.: 20/1119). The TMA includes 34 Congo red-positive biopsies (2 mm diameter) from a total of 18 patients with clinically diagnosed amyloidosis subtypes. All patients gave written informed consent for the use of their tissues for research purposes. Before inclusion, all patient data were pseudonymized. The biopsies were collected from different tissue types, including skeletal muscle, lung, salivary gland/soft tissue, liver, amyloidoma, and kidney. The tissue type distribution of the TMA is shown in Figure S1. The represented amyloidosis subtypes include transthyretin (ATTR) amyloidosis, immunoglobulin light chain amyloidosis (AL; κ and λ), and the secondary AA amyloidosis (serum amyloid A (SAA)). The amyloidosis subtype was retrieved from clinical records. For two cores (NA), this information was not available.

### Sample preparation for MALDI imaging of tryptic peptides

Sample preparation was carried out as published previously [20]. In short, two adjacent tissue slides were cut from the FFPE tissue block and subjected to MALDI imaging measurement of tryptic peptides. Subsequently, these two similarly prepared tissue slices were eventually used for explorative MS1 imaging and iprm-PASEF measurement. Deparaffinization was performed using xylol and ethanol/water dilutions. The sections were washed with ammonium bicarbonate solution and underwent antigen retrieval in a citric acid buffer at ~ 100 °C for 1 h in a steamer. After drying, trypsin (0.1 mg/mL in 10% ACN and 40 mM ammonium bicarbonate) was sprayed onto the slides using an M3 + sprayer (HTX Technologies, Chapel Hill, NC, USA), followed by incubation at 50 °C for 2 h. Alpha-cyano-4-hydroxycinnamic acid matrix (10 mg/mL in 70% ACN, 7 mM ammonium phosphate, and 0.1% TFA) was then applied using the same sprayer device. The spraying parameters were set according to [20].

### MALDI TIMS MS1 imaging acquisition, data processing and data analysis

MALDI TIMS MS1 imaging was performed as published previously [20]. In short, MALDI TIMS MS1 imaging acquisition was performed using a timsTOF fleX mass spectrometer (Bruker Daltonics, Bremen, Germany) in positive-ion reflector mode with a 50 × 50 µm pixel size, 600 shots per pixel, and a 10 kHz laser frequency. Tuning parameters were as published [20]. The data was analyzed using SCiLS Lab software (Bruker Daltonics, Bremen, Germany), with an *m*/*z* interval width of 10 ppm and 0.01 V·s/cm^2^ for 1/*K*_0_. Root mean square normalization was applied. Feature finding was performed with T-ReX3 using spatial smoothing (2 × 2), 50% coverage, and a 1% relative intensity threshold. Manual peak selection was used when necessary.

### Literature search of representative peptide sequences for iprm-PASEF panel design

As a foundation of the iprm-PASEF panel, we sought to retrieve experimentally derived *m*/*z* values for peptides of amyloidogenic proteins as determined by MALDI-MS. Literature search was performed by searching PubMed and Google Scholar in 12/2024 using the following keywords: “MALDI imaging peptide amyloidosis,” “MALDI MSI peptide imaging amyloid deposits amyloidosis,” “SAA amyloidosis MALDI imaging,” and “AL amyloidosis MALDI imaging.” Ultimately, eight publications were screened for *m*/*z* values with annotated peptide identifications derived from amyloid-associated and amyloidogenic proteins [16–19, 21–24]. We focused on publications that included MALDI in situ MS/MS for peptide identification and did not consider publications without a clear description of the identification method (Table S1).

### iprm-PASEF acquisition, data processing and data analysis

As described previously [20], the iprm-PASEF dataset was acquired using the iprm-PASEF tool for timsControl 6.0.8, provided by Bruker Daltonics. Measurements were performed in prm-PASEF and positive-ion reflector mode on a timsTOF fleX with a 100× 100 µm pixel size, 2000 shots per pixel, and a 5 kHz laser frequency. The *m*/*z*-based isolation and fragmentation settings were chosen as prm-PASEF settings. The *m*/*z* range was 100–2,000, and the 1/*K*_0_ range was 1.2–2.1 V·s/cm^2^ with a 230 ms ramp time. Optimized tuning parameters included collision RF (750 Vpp), ion energy (5 eV), transfer time (55 ms), and pre-pulse storage (8 µs). TIMS cell pressure and 1/*K*_0_ calibration followed standard MALDI TIMS MS1 imaging procedures. The iprm-PASEF method targeted nine precursor ions with predefined 1/*K*_0_ and *m*/*z* ranges, using collision energies from 45 to 140 eV based on precursor mass. Thus, obtained MS2 spectra from the iprm-PASEF datasets were averaged across all pixels in DataAnalysis software and exported as “.mgf” files. These were submitted to the MASCOT MS/MS Ions Search against the Swiss-Prot database (April 2024) for *Mus musculus* and *Homo sapiens*. Trypsin digestion with up to one missed cleavage was allowed, with a precursor tolerance of 100 ppm, fragment ion tolerance of 0.3 Da, and a 1% FDR. Searches were performed with and without the variable modifications of oxidation of histidine, tryptophan, proline, and methionine. MS2 spectra and theoretical b- and y-ions (matched within 0.3 Da) were visualized using pyteomics in Python [25]. Peptide fragment ion images were plotted in SCiLS Lab.

### LC-ESI-TIMS-MS/MS sample preparation, acquisition and data analysis focused on singly charged peptides

As previously described [20] after MALDI imaging, co-crystallized peptides were extracted by dissolving the matrix layer in 70% acetonitrile, followed by vacuum drying and peptide cleanup using PreOmics Cartridges. The cleaned peptides were dissolved in water with 0.1% formic acid and desalted on Evotips before LC-ESI-TIMS-MS/MS analysis. LC-ESI-TIMS-MS/MS was performed on a timsTOF fleX mass spectrometer with a CaptiveSpray ion source, using the Evosep One HPLC system with a 44-min gradient and a reversed-phase column. Samples were analyzed in positive data-dependent acquisition (DDA)-PASEF mode, with a mass range of 100–1700 m/*z* and a 1/*K*_0_ range of 0.7–2.1 V·s/cm^2^. The TIMS cell pressure and ion mobility calibration were adjusted to enhance the detection of singly charged peptides. Key tuning parameters were optimized, including a capillary voltage of 1600 V, drying gas flow of 3 L/min at 180 °C, and collision energy of 20–75 eV.

Data analysis was performed using the FragPipe pipeline (v22.0). MS2 peptide identification was done with MSFragger, setting hydroxylation (+ 15.999 Da) of proline as a variable modification and using Bruker.d raw data as input. Human protein databases (including contaminants and internal standards) were used, with reversed sequences as decoys.

### Use of large language models, AI, and machine learning tools

“ChatGPT” and “DeepL Write” were used to improve language and writing style. “ChatGPT” was employed to support Python scripting.

## Results

### iprm-PASEF of amyloidosis TMA

#### MS1 imaging acquisition

**Fig. 1:**
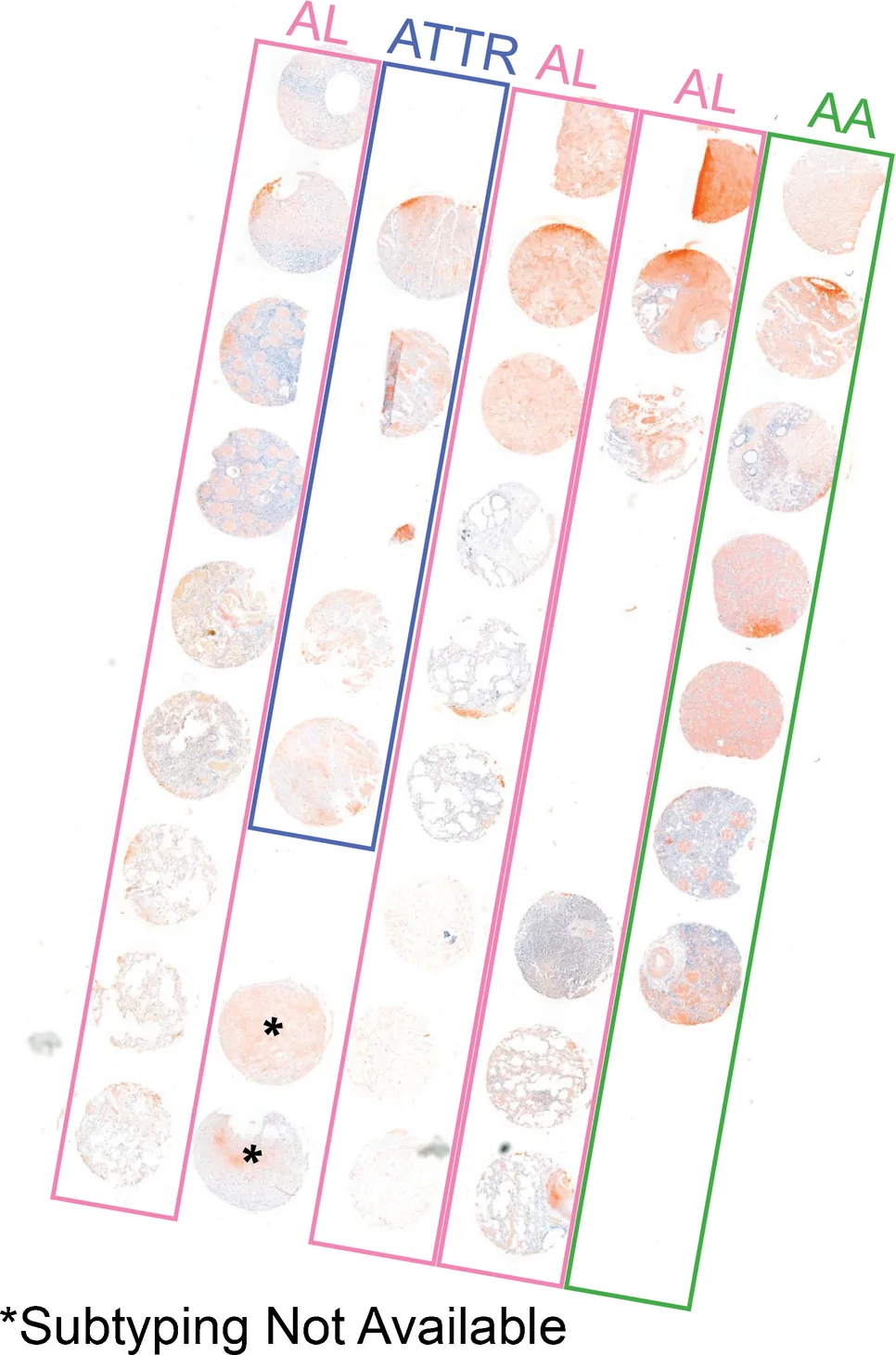
Overview of the amyloidosis TMA composition. Congo red staining of the amyloidosis TMA. The TMA includes 34 biopsies (2 mm diameter) from a total of 18 patients. The represented amyloidosis subtypes include transthyretin (ATTR, blue) amyloidosis, immunoglobulin light chain amyloidosis (AL; κ and λ, pink), and the secondary AA amyloidosis (serum amyloid A (SAA), green)

In this proof-of-concept study, the applicability of iprm-PASEF to identify a small panel of amyloidosis-associated peptides was tested on a TMA comprising tissue samples of different amyloidosis subtypes (ATTR, AL (κ), AL (λ), AA) (Fig. 1). MS1 tryptic peptide imaging (50 × 50 µm spatial resolution) of the amyloidosis TMA comprising 34 tissue cores derived from 18 patients took 230 min and generated 48,311 pixels, resulting in a speed of approximately 3.5 pixels per second, which is less than 200 min per cm^2^ of tissue. A total of 1390 features (MS1 peaks which are defined by a specific *m*/*z* value and a potential 1/*K*_0_ value) were found using the SCiLS Lab Software (Figure S2).

#### Precursor selection for amyloid peptide-targeting iprm-PASEF panel design

**Fig. 2:**
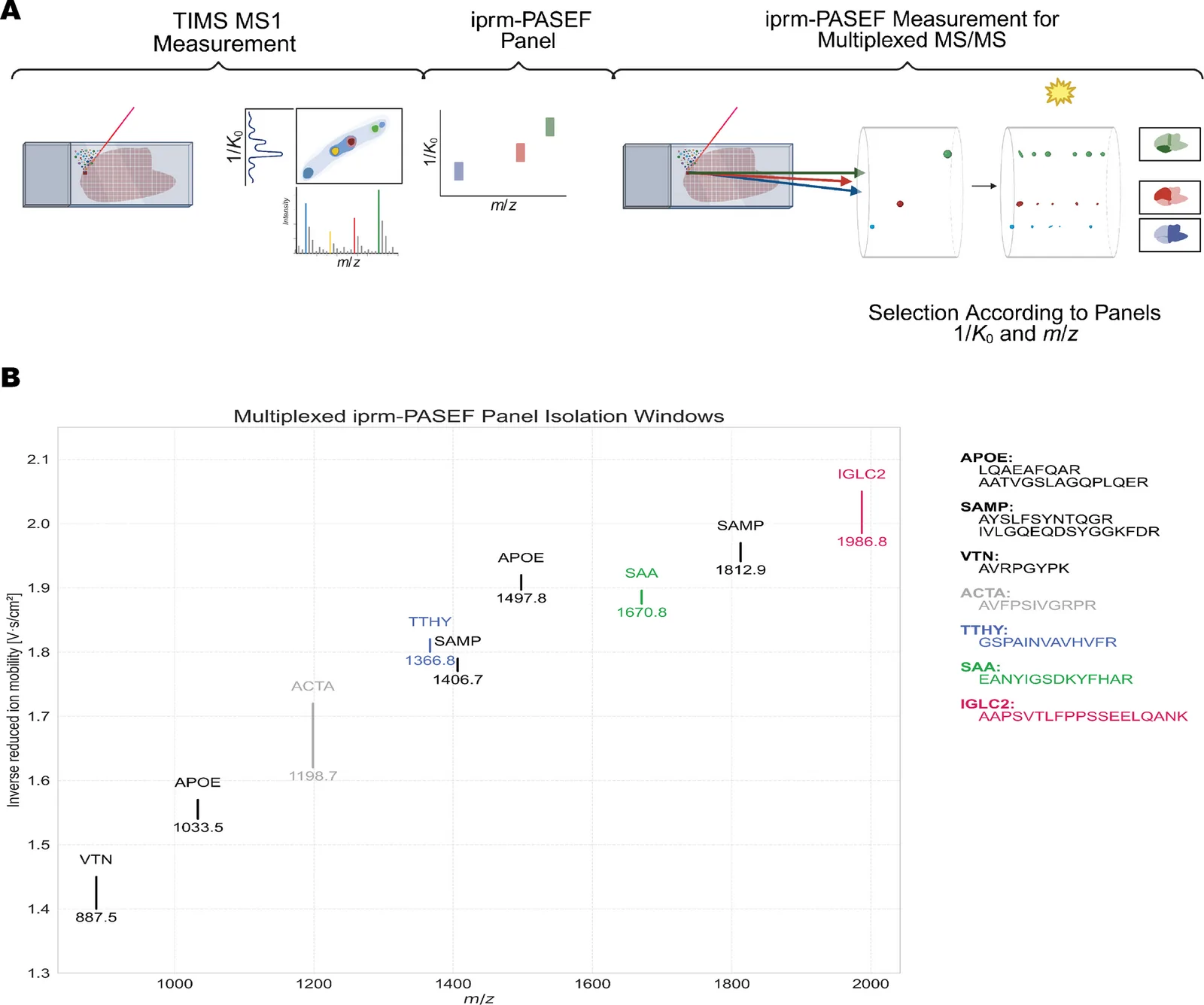
Isolation windows of selected precursors for the multiplexed iprm-PASEF panel.** A** Overview of the iprm-PASEF method used for this study. **B** The *m*/*z* and ion mobility windows for all selected precursors are visualized, highlighting the usage of the entire available ion mobility range

The iprm-PASEF tool allows multiplexed and efficient MS/MS acquisition in MALDI imaging (Fig. 2A). However, it is restricted to a maximum of 15 precursors in one measurement when using the m/z-based isolation and fragmentation mode. Therefore, it is necessary to shorten the list of 1390 features identified in the MS1 measurement. To accomplish this, an iprm-PASEF panel was designed that can identify and characterize amyloidosis-diseased tissue in a single MALDI imaging measurement. To achieve this, the literature was searched to compile a list of potential m/z values for analyzing amyloid-associated and amyloidogenic proteins and their respective peptides as described in detail in Sect. “Literature Search of Representative Peptide Sequences for iprm-PASEF Panel Design.” Only publications that performed tryptic peptide MALDI imaging and employed an in situ MS/MS method for peptide identification were included. We chose a literature search over, e.g., internal LC–ESI–MS/MS analyses because, in our experience, MALDI-based peptide identifications were more reproducible due to peptide flyability and coverage differences between MALDI and ESI. Furthermore, we opted for a first proof-of-concept establishment of a panel targeting amyloidosis-derived peptides rather than for the identification of novel amyloidosis-related features. A detailed list can be found in Table S1. Each collected *m*/*z* value was further investigated in the MS1 imaging dataset by signal intensity and distinct ion image distribution. In addition, signals were examined for overshadowing by high-intensity signals (including isotopic signals) derived from structural proteins not associated with amyloidosis, such as collagen or actin. For this purpose, the ion images were compared with those of known collagen or actin peaks (e.g., *m*/*z* 836.4425 [26, 27] or *m*/*z* 1105.5731 [26, 28] for collagen and *m*/*z* 1198.7024 [29] for actin A) and ion images similar to those of collagen and actin-associated peaks were excluded as potential precursors of interest. Thereby, the *m*/*z* list obtained from literature was further refined. Of note, we later show how the presented iprm-PASEF methodology separates MS2 information of collagen-associated peaks, hence background noise, from other precursors of interest, in our example of APOE and VTN-derived peptides (Sect. “MS/MS Imaging By iprm-PASEF”). As the multiplexed iprm-PASEF requires non-overlapping 1/*K*_0_ windows and the precursor ion entries are presently limited by the software to 15 for the *m*/*z*-based isolation and fragmentation, the number of selectable precursors is restricted. Hence, we aimed to cover amyloid-associated proteins with at least one peptide, simultaneously targeting as many different proteins as possible within the restriction by isolation window availability. Additionally, one precursor at *m*/*z* 1198.7024 (frequently reported as actin A (ACTA) in literature [29]) was included due to the availability of isolation window area as a positive control for the measurement. The resulting precursor list and selected isolation windows can be found in Fig. 2B.

We are aware that literature-based peptide annotations provide only limited confidence, mainly due to the inability of MS1* m*/*z* values alone to distinguish between isobaric peptides. Therefore, a subsequent iprm-PASEF measurement was performed to confirm correct peptide identities by in situ MS/MS directly on the tissue of interest and after ion mobility separation.

#### MS/MS imaging by iprm-PASEF

The iprm-PASEF measurement was performed with a pixel size of 100 × 100 µm on an adjacent TMA slide using the aforementioned precursor list (Fig. 2B). The measurement generated 13,667 pixels in 112 min, resulting in approximately 2 pixels per second, which is less than 100 min per cm^2^ of tissue**.** Bruker’s raw data was imported into DataAnalysis (Bruker Daltonics, Bremen, Germany) and automatically split according to precursor-specific ion-mobility traces. The resulting mean MS2 spectra were exported in a.mgf format and submitted to the online MS/MS ion search engine from MASCOT, allowing for peptide identification. In total, eight out of nine precursors from the iprm-PASEF measurement were successfully identified by MASCOT. The MASCOT score, provided by Matrix Science, statistically evaluates peptide-to-spectrum matching (PSM) confidence, with higher scores indicating greater reliability. In this study, scores ranged from 18.62 to 44.19, with a mass accuracy lower than 35 ppm, consistent with those reported in previous in situ MALDI MS/MS studies [30–33].

Interestingly, two additional features at *m/z* 886.4770 and *m/z* 886.4414 were co-isolated with the precursor at *m/z* 887.5069 (identified as VTN). The fragment signals were separated by their ion mobility trace, and three distinct MS2 spectra were extracted and analyzed successfully by MASCOT (Figure S3) as peptides assigned to COL1A1 and APOE and are included in Table 1. The *m/z* isolation widths in many standard DDA measurements in LC–MS/MS are in the range of 2 Da. In LC–MS/MS, chromatographic peptide separation largely alleviates chimeric MS2 spectra stemming from co-isolated precursors. For mass spectrometry imaging, this example illustrates how separation and enrichment along the CCS-dimension alleviates the co-isolation of precursors for fragmentation to the extent that interpretable MS2 spectra are obtained [34, 35]. 

The isolation window targeting an immunoglobulin light chain-derived peptide at *m*/*z* 1986.8565 and 2.0179 V·s/cm^2^ isolated an MS2 spectrum that includes the fragment signal at *m*/*z* 175.1186, indicating arginine as a y1-ion. However, we were not able to identify the isolated tandem mass spectra by MASCOT, and the identity remains elusive.

In summary, we identified 10 peptides in one single iprm-PASEF run by using the MASCOT search engine (Table 1).

**Table 1:** Validations of peptide identities detected in iprm-PASEF of an amyloidosis TMA. *NI* not identified

Protein name	UniProt ID	Peptide sequence	MASCOT score (higher better)	Mass accuracy (ppm)	Measured* m/z* value	Measured 1/*K*_0_ (V•s/cm^2^)	Number of fragment ion matches
Apolipoprotein E	P02649	ELQAAQAR	29.41	30.68	886.4770	1.4096	10
Collagen 1 A1	P02452	GSEGPQGVR	27.70	1.46	886.4414	1.3907	6
Vitronectin	P04004	AVRPGYPK	41.55	6.09	887.5069	1.4607	7
Apolipoprotein E	P02649	LQAEAFQAR	28.06	17.32	1033.5419	1.5689	6
Actin A	P62736	AVFPSIVGRPR	33.52	6.92	1198.6941	1.7074	9
Transthyretin receptor	P02766	GSPAINVAVHVFR	25.76	4.39	1366.7593	1.8016	8
Serum amyloid P component	P02743	AYSLFSYNTQGR	18.62	3.77	1406.6599	1.7915	11
Apolipoprotein E	P02649	AATVGSLAGQPLQER	43.22	0.33	1497.7979	1.8963	16
Serum amyloid A	P0DJI8	EANYIGSDKYFHAR	44.19	1.80	1670.7859	1.8736	9
Serum amyloid P component	P02743	IVLGQEQDSYGGKFDR	34.88	0.94	1811.8796	1.9492	10
Immunoglobulin light chain 2	P0DOY2	NI	NI	NI	NI	NI	NI

#### Further corroboration of peptide identities

**Fig. 3:**
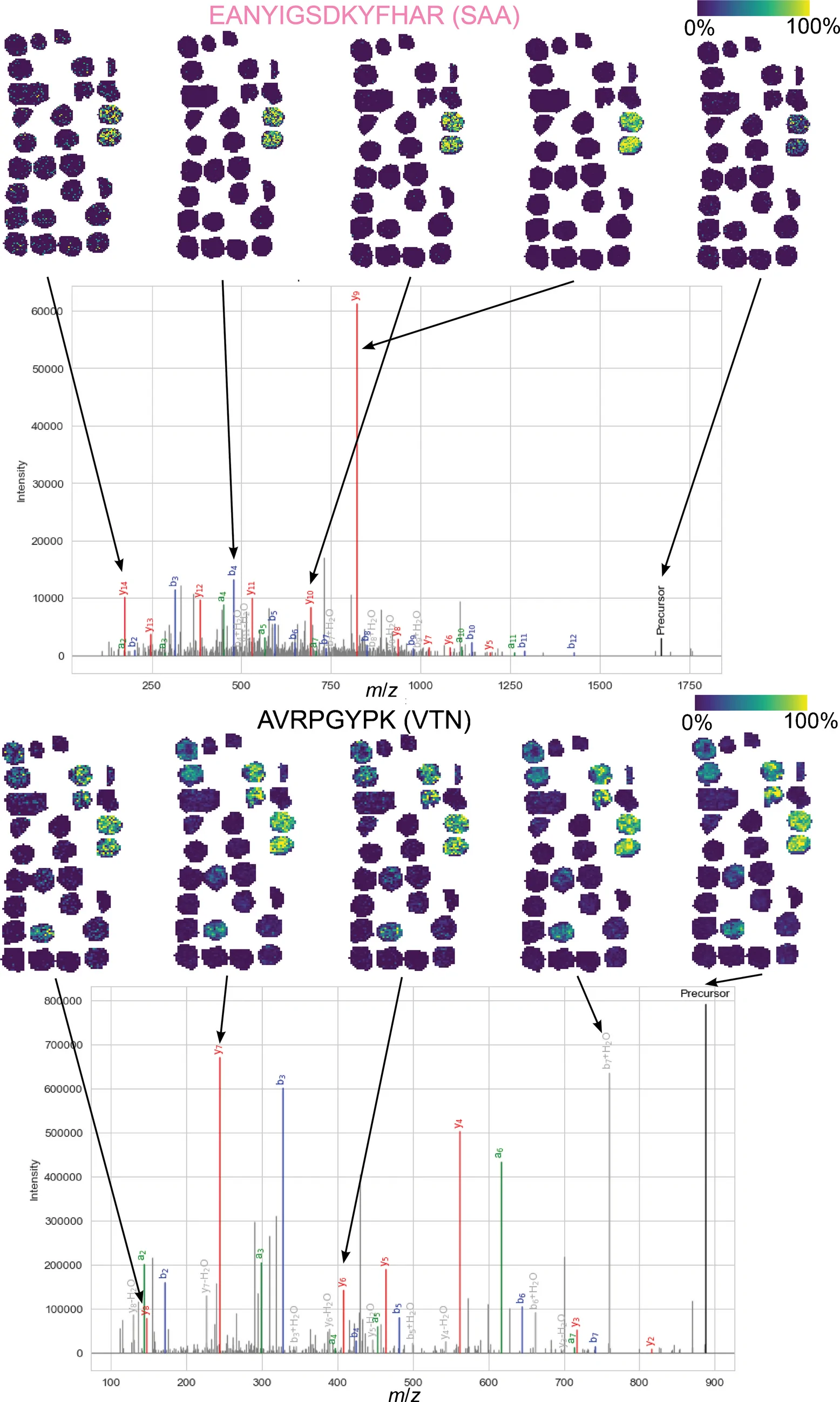
MS2 spectrum of the targeted SAA peptide EANYIGSDKYFHAR. Mean MS2 spectrum acquired in iprm-PASEF with a spatial resolution of 100 µm is shown for two selected precursors (SAA and VTN) of the amyloidosis TMA. B-ions are highlighted in red and y-ions in blue. Ion images of respective fragments are shown on top of the spectrum

We further sought to corroborate the peptide identities obtained by iprm-PASEF and MASCOT on the spatial level. To this end, we annotated the predicted b- and y-ions of the mean MS2 spectra with the pyteomics tool [25]. All mean spectra can be found in the Supplementary Information (Figure S4). To further corroborate peptide sequence identification by MS2, we used synthetic peptides of four selected precursors (VTN_AVRPGYPK, APOE_LQAEAFQAR, TTHY_GSPAINVAVHVFR, SAMP_AYSLFSYNTQGR) to generate reference MS2 spectra applying the same iprm-PASEF instrument tuning parameters. Fragment ion detection and respective intensity patterns were comparable and all MS2 spectra can be found in the Supplementary Information (Figure S5). Further, the co-localization of matched b- and y-ions to the respective precursor was investigated in SCiLS Lab to assess the spatial distribution of the MS/MS information. We visually assessed the co-localization of the targeted precursors with their expected b- and y-ions in the MS2 ion images (difference of maximum 15 ppm). For all precursors, except TTHY, we were able to find at least four fragment ion images which co-localized with their respective precursor (Figure S6). For example, the precursor with the peptide sequence EANYIGSDKYFHAR and the *m*/*z* value of 1670.7859 showed a similar spatial distribution to its matched fragment ions (Fig. 3 top). Another example is the precursor with the peptide sequence AVRPGYPK and the *m*/*z* value of 887.5069, which also showed a similar spatial distribution to its matched fragment ions (Fig. 3 bottom).

To further corroborate peptide identification, residual matrix after iprm-PASEF was used to extract peptides for LC-ESI-TIMS-MS/MS measurement. The measurement method included singly charged peptides to allow for the comparison on 1/*K*_0_ ion mobility values in addition to *m*/*z* values. Nine out of ten iprm-PASEF identified peptides could be detected in LC–ESI–MS/MS with an *m*/*z* variance of less than 10 ppm. Five of them were even detected as singly charged ions, enabling the direct matching of *m*/*z* and 1/*K*_0_ values. The observed 1/*K*_0_ variance between MALDI and ESI was lower than 0.025 V·s/cm^2^ (Table S2).

### iprm-PASEF allows for the in situ, antibody-independent characterization of amyloidosis subtypes

Having established a small-scale iprm-PASEF amyloidosis panel, we investigated to what extent MSI-based characterization of amyloidosis subtypes correlates with their clinical-pathological diagnosis. To this end, the acquired iprm-PASEF dataset was aligned with the clinical annotations extracted from the corresponding medical records. MS1 imaging and iprm-PASEF datasets were co-localized visually with a consecutive Congo red staining.

**Fig. 4:**
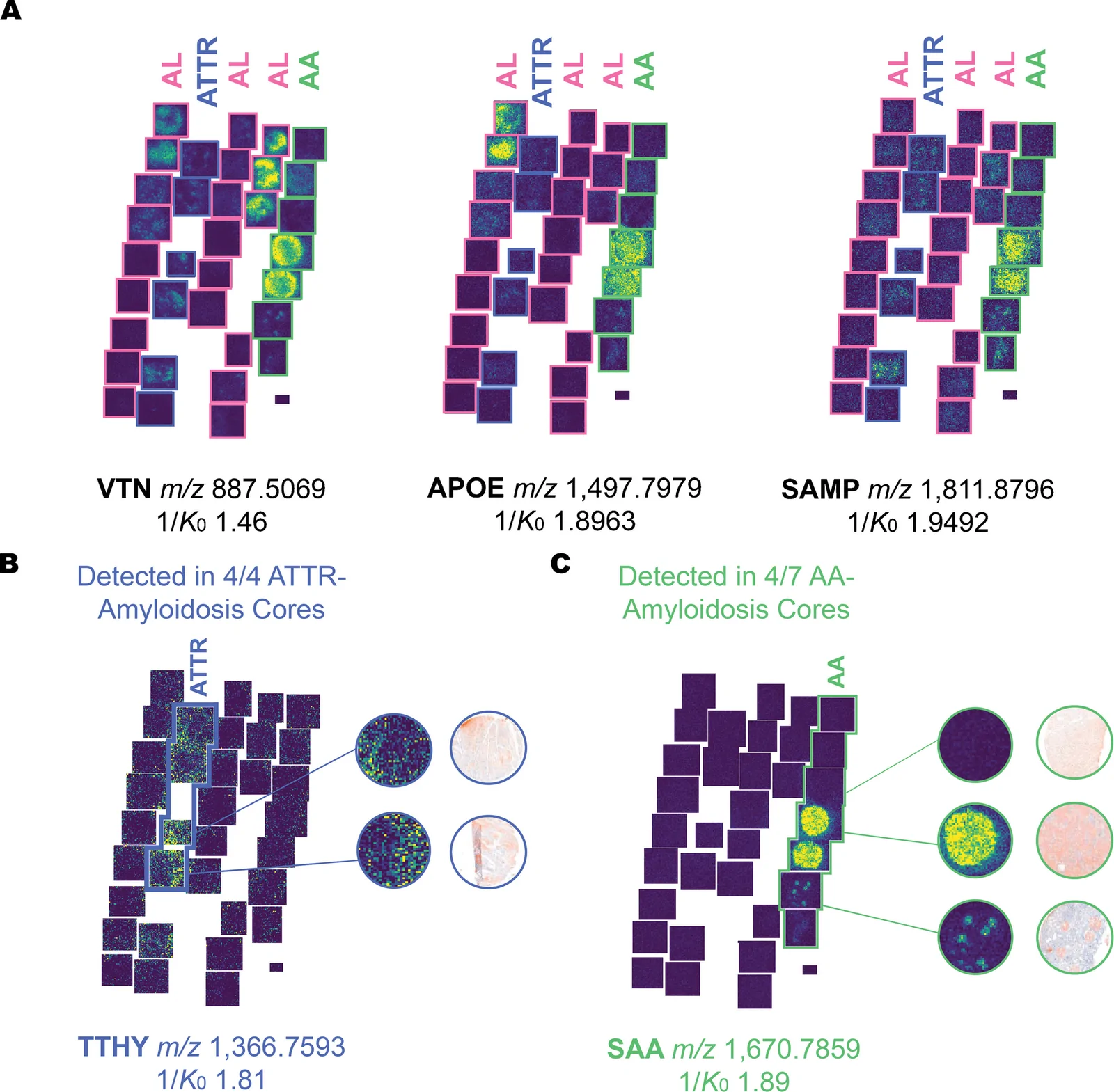
Characterization and subtyping of the amyloidosis TMA by MALDI imaging.** A** Characterization of amyloidosis-related proteins with targeted peptides (APOE, SAMP, VTN). **B** Proteins and their targeted peptides for subtyping ATTR-amyloidosis (TTHY). Congo red staining of some selected cores is visualized for comparison. **C** Proteins and their targeted peptides for subtyping AA-amyloidosis (SAA). Congo red staining of some selected cores was visualized for comparison

As expected, the targeted peptides from the amyloid-associated proteins APOE, SAMP, and VTN were found in all subtypes (Fig. 4A). For instance, SAMP is commonly found in most amyloid cores [3, 5, 6]. In our TMA, we detected two SAMP-derived peptides in varying abundances in amyloid cores of all subtypes. On the other hand, the protein APOE is usually found at low levels in some AA and AL amyloidosis cores [3–5]. In this study, peptides from APOE were found in varying abundances in AA and AL amyloidosis cores. Most importantly, we identified several APOE and SAMP peptides with a similar location within the tissue, confirming that the spatial resolution of the protein remains intact even after digestion into peptides. We further targeted one peptide of the protein VTN that is reported to be present in most amyloidosis subtypes [3]. Within our TMA, it was detected with differing abundance in cores of all subtypes (Fig. 4A, Figure S7).

Regarding the targeting of amyloidosis subtype specific peptides, we identified the peptide GSPAINVAVHVFR (residues 42–54 of TTHY) by iprm-PASEF with low intensity, indicating weak expression of the protein TTHY in all four ATTR-amyloidosis cores. The peptide was detected below noise level in the cores assigned to the other subtypes AL and AA (Fig. 4B, Figure S7). Notably, this TMA contains two amyloidosis cores for which subtyping is unknown. We detected the TTHY expression in one of these cores, which suggests that ATTR may be the underlying subtype.

Meanwhile, the subtype-specific peptide EANYIGSDKYFHAR (residues 44–57 of SAA) was identified in four out of seven AA-amyloidosis cores with varying intensity, while remaining absent in AL and ATTR cores showing high specificity for AA amyloidosis. Most interestingly, the Congo red-positive area in these SAA-containing cores shows close alignment with the ion image of the SAA peptide detected by the iprm-PASEF panel with MALDI imaging (Fig. 4C, Fig S5). For the AL-amyloidosis subtype, the IGLC2 peptide could not be identified within the presented iprm-PASEF setup and awaits further investigation.

In total, we identified nine peptides derived from five different amyloid-related proteins by applying our designed iprm-PASEF panel. Thereby, heterogenous amyloidosis-diseased tissues were spatially characterized by their composition of subtype-specific and amyloid-associated proteins.

### TIMS-enabled separation of isobaric signals

By the inclusion of the ion mobility, TIMS-included MALDI imaging measurements can lead to an increased signal resolution and enable deconvolution of features, e.g., as shown for the co-isolated peptides at m/z 887.5069 for the iprm-PASEF panel and as shown in further published studies [36, 37].

**Fig. 5:**
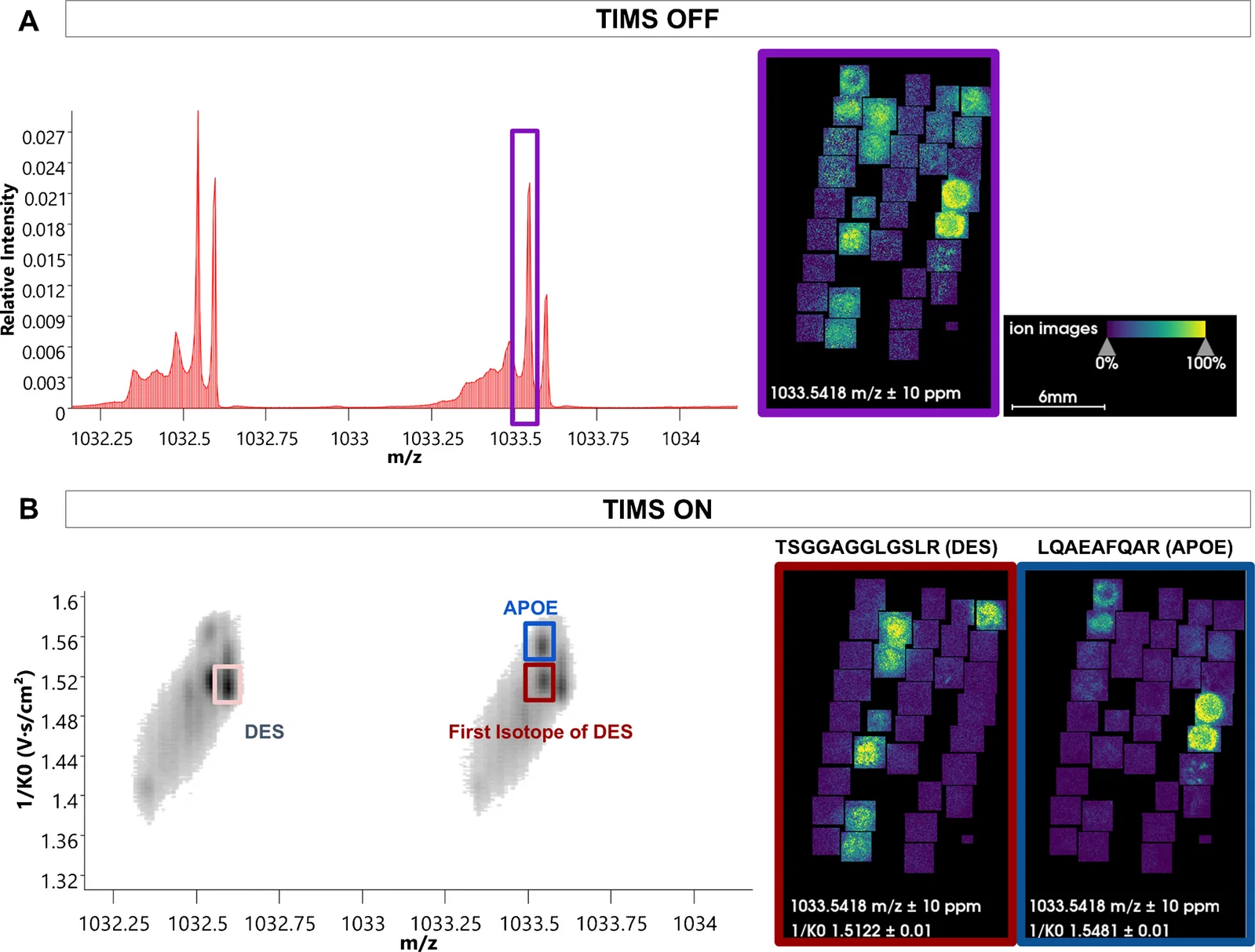
TIMS-enabled separation of isobaric signals.** A** Peak at *m*/*z* 1033.5 unfiltered for ion mobility shows a diffuse localization over the whole tissue. **B** By including the TIMS dimension, a second precursor is noticed underlying the *m*/*z* 1,033.5 peak with two different ion mobilities (1.51 V·s/cm^2^ and 1.54 V·s/cm^2^). When targeting the peaks with iprm-PASEF, APOE and the M + 1 peak of DES were identified. The ion images show a much clearer localization when the TIMS dimension is included in the measurement

When looking closer at the MS1 signal detected at *m*/*z* 1033.5418 in our experiment, we noticed that the *m*/*z* peak was divided into two signals in the ion mobility dimension with two different 1/*K*_0_ values (1.51 V·s/cm^2^ and 1.54 V·s/cm^2^). Interestingly, those two signals showed divergent ion images, indicating that the peak is derived from two distinct isobaric molecules and the mobility difference is not solely caused by conformational change (Fig. 5). The signal at 1.54 V·s/cm^2^ was assigned to LQAEAFQAR from APOE (Mascot score 28) in the iprm-PASEF measurement (3.1.3). To further investigate the identity of *m*/*z* 1033.5418 at 1.51 V·s/cm^2^, we used the iprm-PASEF mode with only two isolation windows (m/z range for both: 1031.00–1035.00; ion mobility windows: 1.50–1.522 and 1.53–1.56 V·s/cm^2^) for MS/MS identification in an additional measurement, revealing the signal at 1.51 V·s/cm^2^ as the M + 1 peak of *m*/*z* 1032.5418, detected at *m*/*z* 1033.5418, and identified as TSGGAGGLGSLR from desmin (DES, P17661, Mascot score 45).

This result underscores the advantage of incorporating the TIMS dimension into MALDI imaging of tryptic peptides; in the MS1 MALDI TIMS imaging, *m*/*z* 1033.5418, referred to as APOE signal in literature, would have compiled a background signal derived from desmin, possibly leading to a false positive result for APOE in certain cores, as illustrated in the ion images in Fig. 5. This also highlights the advantage of full spatial coverage for MS/MS acquisition. Overall, by incorporating the TIMS dimension, we were able to successfully isolate the APOE signal at *m*/*z* 1033.5418 from interfering signals, thereby improving both the resolution and reliability of the peptide MALDI imaging measurements.

## Discussion

In this study, we present a novel iprm-PASEF panel for characterizing the protein composition of amyloid plaques in situ. This proof-of-concept study identified a total of ten peptides derived from seven proteins, in particular five proteins related to amyloidosis disease, in a single MALDI imaging measurement and visualized their spatial distribution. Furthermore, we have demonstrated the advantage of including the TIMS dimension into MALDI imaging measurements, thereby separating isobaric signals and performing iprm-PASEF for identification.

In our iprm-PASEF panel, we targeted a total of three peptides derived from subtype-specific proteins (SAA, IGLC2, and TTHY). Two of those peptides were successfully identified within their respective clinical subtypes (AA and ATTR), with variability observed between patients and tissue types. We manually evaluated the spatial similarities between the identified peptides and the Congo red staining. In the future, this step could be performed with a less biased approach, such as by applying an automated co-registration pipeline [38]. The significant variability observed among patients and tissue types may be explained by the varying accessibility of heat-induced antigen retrieval to different tissue structures or inefficient trypsin digestion [39]. We failed to identify the third targeted subtype-specific peptide (IGLC2), which is usually found in AL amyloidosis; this is despite its identification by additional LC–ESI–MS/MS measurements. We speculate that the underlying proteins have too low abundance in the presented TMA to be detected and identified correctly. Further studies and possibly AL-dedicated iprm-PASEF panels may be required to yield their identification in MALDI imaging.

All five amyloidosis-associated peptides included in the iprm-PASEF panel could be successfully identified. SAMP and APOE were covered with up to two peptides per protein that all showed a high alignment in spatial resolution within one protein species. Although this approach was important for confirming the spatial localization of proteins after digestion, future approaches could replace these targets with other precursors of interest, e.g., other peptides specifically derived from AL-amyloidosis. This would optimize the use of the limited multiplexing capacity per acquisition cycle (approximately ten), since maintaining non-overlapping 1/*K*_0_ isolation windows is currently still necessary. One way to address this issue and thereby increase the number of targetable precursors per scan is to use a raster offset to schedule multiple isolation window panels. This would allow the same tissue sample to be acquired repeatedly. Alternatively, one could acquire in situ MS/MS information spotwise and automatically using the spatial ion mobility-scheduled exhaustive fragmentation (SIMSEF) tool [40]. This approach has the potential to significantly increase the number of precursors submitted for MS/MS fragmentation in a single run but comes at the cost of strongly reduced spatial MS/MS coverage.

The presented workflow can be further improved by establishing dedicated software for analyzing iprm-PASEF datasets. As outlined in our previous publication, our data analysis pipeline relied on a combination of several different tools, thereby entering multiple import and export steps that introduce inefficiencies and potential sources of error [20]. In addition, manual adjustments were necessary, for example, due to the limited performance of the SCiLS Lab feature detection tool for 1/*K*_0_ dimensional data. Furthermore, this study only used the mean MS2 spectrum across the entire tissue region for peptide identification, which could potentially introduce a significant noise level. Integrating precursor-specific regions could substantially improve the quality of the MS2 spectra, resulting in more reliable identification. Additionally, MS2 spectrum quality is highly dependent on the mobilogram region selected for integration in DataAnalysis, indicating that multiple peptide species may have been co-isolated within a single window. Therefore, by automating the data analysis pipeline and using more sophisticated and adapted software, e.g., for PSM, identification and throughput could be increased.

In conclusion, this study applies an amyloidosis-targeting iprm-PASEF panel to a TMA derived from 18 patients, thus identifying and spatially mapping amyloidosis-associated peptides in a multiplexed manner. This allows for a more refined characterization of Congo red-positive amyloid plaques. It further opens novel possibilities for the usage of MALDI imaging in spatial proteomics.

## Supplementary Information

Below is the link to the electronic supplementary material.

Supplementary Material 1 (PPTX 20.3 MB)

Supplementary Material 2 (PDF 525 KB)

Supplementary Material 3 (PDF 418 KB)

## Data Availability

The raw data are publicly available on MassIVE (Mass Spectrometry Interactive Virtual Environment) under the following links: Amyloidosis MS1 data: 10.25345/C5SJ1B45W] (MSV000099481) Amyloidosis MS/MS data: 10.25345/C5NS0MB27 (MSV000099482)

## Abbreviations

ACTA: Actin A
APOE: Apolipoprotein E
FFPE: Formalin-fixed paraffin-embedded
IGLC2: Immunoglobulin Light Chain 2
iprm-PASEF: Imaging Parallel Reaction Monitoring, Parallel Accumulation SErial Fragmentation
LC–ESI–MS/MS: Liquid-chromatography electrospray ionization tandem mass spectrometry
LCM: Laser capture microdissection
MALDI: Matrix-assisted laser desorption/ionization
MSI: Mass spectrometry imaging
prm: Parallel reaction monitoring
PASEF: Parallel Accumulation SErial Fragmentation
PSM: Peptide to spectrum matching
SAA: Serum amyloid A
SAMP: Serum amyloid P component
TIMS: Trapped Ion mobility spectrometry
TMA: Tissue microarray
VTN: Vitronectin
1/*K*_0_: Inverse of reduced ion mobility
