# Supplementary material for: In situ, antibody-independent, and multiplexed characterization of amyloid plaques by MALDI MS/MS imaging using iprm-PASEF
Source: Anal Bioanal Chem. 2026 Jan 14;418(20):6633–46. doi: 10.1007/s00216-025-06277-1 (PMC13619668; doi:10.1007/s00216-025-06277-1)
Supplement: Supplementary file 1 [file 216_2025_6277_MOESM2_ESM.pdf]

m/z	Protein name	Peptide	Identification method	paper	Lme048 Signal detected?
875,47	VTN		LC-MS/MS	https://doi.org/10.1007/s00216-015-8689-z	no
883,47	IGLV2-11			doi:10.1136/jcp-2023-208790	no
887,51	Vitronectin	AVRPGYPK	On tissue MS/MS MALDI	DOI: 10.1002/pmic.201700236	yes
887,51	VTN		LC MS/MS	https://doi.org/10.1007/s00216-015-8689-z	yes
948,526	Apolipoprotein E	LAVYQAGAR	MALDI MS/MS	DOI: 10.1369/0022155415595264	yes
948,53	Apolipoprotein E	LAVYQAGAR	On tissue MS/MS MALDI	DOI: 10.1002/pmic.201700236	yes
948,539	APOE		? no sequence given	https://doi.org/10.1186/s12014-024-09500-w	yes
968,51	IGLV2-11		not stated	DOI: 10.1136/jcp-2023-208790	APOE is overshadowing, only signal of APOE most likely (looking at ion images)
968,55	Apolipoprotein E	LGPLVEQGR	On tissue MS/MS MALDI	DOI: 10.1002/pmic.201700236	yes
968,55	APOE		MALDI MS/MS and LC-MS/MS	https://doi.org/10.1007/s00216-015-8689-z	yes
968,553	Apolipoprotein E	LGPLVEQGR	MALDI MS/MS	DOI: 10.1369/0022155415595264	yes
983,588	Apo A4		IMs orbitrap, LC-MS/MS, and MS-Digest	https://doi.org/10.1371/journal.pone.0275993	
1031,52	ApoAI	LSPLGEEMR	On tissue MS/MS MALDI	DOI: 10.1002/pmic.201700236	no
1033,54	APOE		LC-MS/MS	https://doi.org/10.1007/s00216-015-8689-z	yes
1033,632	APO E			https://doi.org/10.1371/journal.pone.0275993	yes
1074,5	IGL	YASWYQQK	MALDI MS/MS	doi: 10.1016/j.clinbiochem.2013.05.063.	no
1156,6	SAMP		LC-MS/MS	https://doi.org/10.1007/s00216-015-8689-z	yes
1156,606	SAMP		? no sequence given	https://doi.org/10.1186/s12014-024-09500-w	yes
1156,67	Serum amyloid P-component	VGEYSLYIGR	On tissue MS/MS MALDI	DOI: 10.1002/pmic.201700236	yes
1158,58	IGKV2-29			doi:10.1136/jcp-2023-208790	yes
1158,58	VTN		LC-MS/MS	https://doi.org/10.1007/s00216-015-8689-z	s.o.
1166,58	SAMP		LC-MS/MS	https://doi.org/10.1007/s00216-015-8689-z	
1242,69	IGVL325			doi:10.1136/jcp-2023-208790	no
1268,675	TTR	VEIDTKSYWK (expasy 2 missed cleavages allowed)	? no sequence given	https://doi.org/10.1186/s12014-024-09500-w	strong collagen peak overshadowing
1281,647	TTR		? no sequence given	https://doi.org/10.1186/s12014-024-09500-w	no
1301,65	ApoAI	THLAPYSDELR	On tissue MS/MS MALDI	DOI: 10.1002/pmic.201700236	no
1314,68	Vitronectin	RVDTVDPPYPR	On tissue MS/MS MALDI	DOI: 10.1002/pmic.201700236	yes
1314,68	VTN		LC-MS/MS	https://doi.org/10.1007/s00216-015-8689-z	
1314,682	Vitronectin	RVDTVDPPYPR	MALDI MS/MS	DOI: 10.1369/0022155415595264	yes
1366,5	TTR	GSPAINVAVHVFR	MALDI MS/MS	doi: 10.1016/j.clinbiochem.2013.05.063.	no
1366,76	TTR	GSPAINVAVHVFR	On tissue MS/MS MALDI	DOI: 10.1002/pmic.201700236	no
1366,795	TTR		IMs orbitrap, LC-MS/MS, and MS-Digest	https://doi.org/10.1371/journal.pone.0275993	
1396,717	TTR		? no sequence given	https://doi.org/10.1186/s12014-024-09500-w	most likely actin or similar cellular peak
1406,667	SAMP		? no sequence given	https://doi.org/10.1186/s12014-024-09500-w	yes
1406,67	Serum amyloid P-component	AYSLFSYNTQGR	On tissue MS/MS MALDI	DOI: 10.1002/pmic.201700236	yes
1406,672	Serum amyloid P-component	AYSLFSYNTQGR	MALDI MS/MS	DOI: 10.1369/0022155415595264	yes
1422,65	Vitronectin	FEDGVLDPDYPR	On tissue MS/MS MALDI	DOI: 10.1002/pmic.201700236	no
1422,65	VTN		LC-MS/MS	https://doi.org/10.1007/s00216-015-8689-z	
1422,661	Vitronectin	FEDGVLDPDYPR	MALDI MS/MS	DOI: 10.1369/0022155415595264	no
1.423	VTN		IMs orbitrap, LC-MS/MS, and MS-Digest	https://doi.org/10.1371/journal.pone.0275993	
1434,12	IGKV 1-17			doi:10.1136/jcp-2023-208790	no
1456,72	SAA		LC-MS/MS	https://doi.org/10.1007/s00216-015-8689-z	
1456,821	SAA		IMs orbitrap, LC-MS/MS, and MS-Digest	https://doi.org/10.1371/journal.pone.0275993	
1494,851	TTR	GSPAINVAVHVFRK	? no sequence given	https://doi.org/10.1186/s12014-024-09500-w	no
1497,772	APOE		? no sequence given	https://doi.org/10.1186/s12014-024-09500-w	yes
1497,8	Apolipoprotein E	AATVGSLAGQPLQER	On tissue MS/MS MALDI	DOI: 10.1002/pmic.201700236	yes
1497,8	APOE		LC-MS/MS	https://doi.org/10.1007/s00216-015-8689-z	
1503,84	Vitronectin	IYISGMAPRPSLAK	On tissue MS/MS MALDI	DOI: 10.1002/pmic.201700236	yes
1524,5	TTR	AADDT WEPFASGK	MALDI MS/MS	doi: 10.1016/j.clinbiochem.2013.05.063.	no
1540,74	IGHA1		LC MSMS	https://doi.org/10.1007/s00216-015-8689-z	
1550,823	SAA		IMs orbitrap, LC-MS/MS, and MS-Digest	https://doi.org/10.1371/journal.pone.0275993	
1612,82	SAA	RGPGGAWAAEAISDAR	On tissue MS/MS MALDI	DOI: 10.1002/pmic.201700236	yes
1612,82	SAA		LC-MS/MS	https://doi.org/10.1007/s00216-015-8689-z	
1612,934	SAA		IMs orbitrap, LC-MS/MS, and MS-Digest	https://doi.org/10.1371/journal.pone.0275993	
1646,81	VTN		LC-MS/MS	https://doi.org/10.1007/s00216-015-8689-z	
1646,815	Vitronectin	DVWGIEGPIDAAFTR	MALDI MS/MS	DOI: 10.1369/0022155415595264	no
1646,82	Vitronectin	DVWGIEGPIDAAFTR	On tissue MS/MS MALDI	DOI: 10.1002/pmic.201700236	no
1666,77	VTN		LC-MS/MS	https://doi.org/10.1007/s00216-015-8689-z	
1666,78	Vitronectin	DWHGVPGQVDAAMAGR	On tissue MS/MS MALDI	DOI: 10.1002/pmic.201700236	yes
1670,79	SAA	EANYIGSDKYFHAR	On tissue MS/MS MALDI	DOI: 10.1002/pmic.201700236	yes
1670,79	SAA		LC-MS/MS	https://doi.org/10.1007/s00216-015-8689-z	
1743,86	IGLC1	YAASSYLSLTPEQWK (expasy)	LC MSMS, SPEED extraction MALDI MS/MS?	DOI:10.1002/prca.202000079	yes
1811,89	Serum amyloid P-component	IVLGQEQDSYGGKFDR	On tissue MS/MS MALDI	DOI: 10.1002/pmic.201700236	yes
1811,89	SAMP		LC-MS/MS and MALDI MS/MS	https://doi.org/10.1007/s00216-015-8689-z	
1811,891	SAMP		no sequence given	https://doi.org/10.1186/s12014-024-09500-w	yes
1925,3	Ig V-III	EVQLLESGGGLVQPGGLR	MALDI MS/MS, I doublechecked: 1st the mass is completely wrc	doi: 10.1016/j.clinbiochem.2013.05.063.	overshadowed by collagen peak
1986,02	IGLC2 P0DOY3	AAPSVTLFPPSSEELQANK (expasy)	LC-MS/MS, SPEED extraction MALDI MS/MS	DOI:10.1002/prca.202000079	no
1406.67	SAMP		LC-MS/MS and MALDI MS/MS	https://doi.org/10.1007/s00216-015-8689-z	

Suplementary Table 1: Amyloidosis peptides identified in literature using MALDI imaging
