# Supplementary material for: In situ, antibody-independent, and multiplexed characterization of amyloid plaques by MALDI MS/MS imaging using iprm-PASEF
Source: Anal Bioanal Chem. 2026 Jan 14;418(20):6633–46. doi: 10.1007/s00216-025-06277-1 (PMC13619668; doi:10.1007/s00216-025-06277-1)
Supplement: Supplementary file 2 [file 216_2025_6277_MOESM3_ESM.pdf]

Suplementary Table 2: Coroboration of peptide identities found using iprm-PASEF with LC-ESI MS/MS

**Protein Name**	**Peptide Sequence**	**measured m/z value MALDI**	**measured 1/*K* 0 MALDI (V•s/cm2)**	**measured m/z value LC**	**measured 1/*K*0 LC (V•s/cm2) (in case of 1+ charge detected)**	**mass error (ppm)**	**1/*K*0 error**
Apolipoprotein E	ELQAAQAR	886,47	1,406	886,476	1,387	6,7684	0,0190
Vitonectin	AVRPGYPK	887,51	1,436	ND	ND	ND	
Colagene 1 A1	GSEGPQGVR	886,44	1,391	886,437	1,369	3,7228	-0,0215
Apolipoprotein E	LQAEAFQAR	1033,54	1,564	1.033,539	1,554	0,9675	0,0103
Actin A/B	AVFPSIVGRPR	1198,70	1,627	1.198,693		5,7563	
Transthyretin receptor	GSPAINVAVHVFR	1366,74	1,806	1.366,741		0,3658	
Serum amyloid P component	AYSLFSYNTQGR	1406,67	1,784	1.406,661	1,808	6,3981	0,0240
Apolipoprotein E	AATVGSLAGQPLQER	1497,80	1,902	1.497,786	1,901	9,1468	0,0011
Serum amyloid A	DGEAGAQGPP[16]GPAGPAGER	1670,79	1,870	1.670,778		7,0626	
Serum amyloid P component	IVLGQEQDSYGGKFDR	1811,87	1,948	1.811,878		4,6361	
